# Identification of Genes for Complex Diseases Using Integrated Analysis of Multiple Types of Genomic Data

**DOI:** 10.1371/journal.pone.0042755

**Published:** 2012-09-05

**Authors:** Hongbao Cao, Shufeng Lei, Hong-Wen Deng, Yu-Ping Wang

**Affiliations:** 1 Department of Biomedical Engineering, Tulane University, New Orleans, Louisiana, United States of America; 2 Center for Genetic Epidemiology and Genomics, School of Public Health, Medical College of Soochow University, Suzhou, P. R. China; 3 Department of Biostatistics and Bioinformatics, Tulane University, New Orleans, Louisiana, United States of America; Central China Normal University, China

## Abstract

Various types of genomic data (e.g., SNPs and mRNA transcripts) have been employed to identify risk genes for complex diseases. However, the analysis of these data has largely been performed in isolation. Combining these multiple data for integrative analysis can take advantage of complementary information and thus can have higher power to identify genes (and/or their functions) that would otherwise be impossible with individual data analysis. Due to the different nature, structure, and format of diverse sets of genomic data, multiple genomic data integration is challenging. Here we address the problem by developing a sparse representation based clustering (SRC) method for integrative data analysis. As an example, we applied the SRC method to the integrative analysis of 376821 SNPs in 200 subjects (100 cases and 100 controls) and expression data for 22283 genes in 80 subjects (40 cases and 40 controls) to identify significant genes for osteoporosis (OP). Comparing our results with previous studies, we identified some genes known related to OP risk (e.g., *‘THSD4’, ‘CRHR1’, ‘HSD11B1’, ‘THSD7A’, ‘BMPR1B’ ‘ADCY10’, ‘PRL’, ‘CA8’,’ESRRA’, ‘CALM1’, ‘CALM1’, ‘SPARC’*, and *‘LRP1’*). Moreover, we uncovered novel osteoporosis susceptible genes (*‘DICER1’, ‘PTMA’*, *etc.*) that were not found previously but play functionally important roles in osteoporosis etiology from existing studies. In addition, the SRC method identified genes can lead to higher accuracy for the diagnosis/classification of osteoporosis subjects when compared with the traditional T-test and Fisher-exact test, which further validates the proposed SRC approach for integrative analysis.

## Introduction

In genomic data analysis, one of the crucial issues is to identify disease susceptible genes from the vast amount of data [1]–[8]. Some genes are related to the diagnosis task, but many are presumably irrelevant [1]. During the past few years, various clustering techniques have been developed to identify subsets of genes significant for diagnosis or classification of diseases [1]–[7]. Among those gene selection methods, a variety of statistical methods were used. For example, Yang *et al.* [1] used forward sequential feature selection (FSFS) method to remove irrelevant SNP data. Soneson *et al.* [2] used Canonical Correlation Analysis (CCA) for joint analysis of gene expression and copy number variations (CNVs). Berger *et al.* [4] developed a generalized singular value decomposition (GSVD) to locate genes with both high variations and high correlations across samples of gene expression changes and CNVs. These methods demonstrated limited success; there has been continuous demand for the development of efficient data integration technique. In this work, we developed a sparse representation based clustering (SRC) method for gene selection, based on multiple features extracted from genomic data. The features we used here were the statistical measurements of the original genomic data, such as the mean and standard deviation *etc.*. We refer the raw genomic data (SNPs or gene expressions) as variables. The variable selection was performed using the features of the original data instead of using the raw data directly. In this work, we employed 5 features in the analysis of the two data sets (see Section 2.3 ‘Features selection’ for the detailed description). Sparse representation or compressive sensing (CS) is a novel statistical method recently developed in statistics and applied mathematics, which has found many successful applications in diverse disciplines. For example, Wright *et al.* proposed a CS based method for face recognition, which showed better accuracy and resistance to noise [8]. We have developed and applied the SRC method for chromosome image classification and showed improved accuracy [9]. In this work, we apply the SRC algorithm to select genes/variables that are significant for OP using joint analysis of two different types of genomic data: gene expression and SNP data. The description of ‘SRC clustering’ algorithm is given in **Supporting Material S1**.

To validate our method, we apply it to the study of osteoporosis, which is a major public health problem over the world [10]. Osteoporosis is characterized by the low bone mineral density (BMD) [11], which leads to increased risk to fragility fracture. Genetic factors play an important role in the pathogenesis of osteoporosis, as evidenced by high heritability (≥50%) of BMD [12]–[14]; however, specific genetic factors both influencing BMD and contributing to the development of osteoporosis are largely uncharacterized.

Identifying genetic factors for osteoporosis is challenging because of the nature of complex genetic determinations, including polygenic determinations, multiple gene-gene interactions, and multiple gene-environment interactions. So far, great attempts have been made to identify osteoporosis risk genes; however, most of them focused on DNA, RNA, or protein levels individually, which were rarely combined or integrated in a statistically rigorous manner to ascertain the importance of certain gene(s) for bone phenotypes [15], [16]. For example, current genome-wide association studies established relationship of gene(s) and phenotypes (e.g., BMD) at DNA level [17], [18] without considering RNA or protein expression, thus lacking an immediate insight on the functions of genes or gene expression regulations. Integrating substantial evidences from different levels (i.e., DNA, RNA and protein) can not only improve chances of identifying genetic factors for osteoporosis, but also ascertain the potential functioning mechanisms of genes and their contributions to osteoporosis.

The paper is organized as follows. We first briefly describe the SRC method and the resulting gene shaving algorithm we proposed. Then we apply the method to gene selection with integrative analysis of both gene expression and SNP data from osteoporosis patients. For the purpose of comparison with individual data analysis, we also performed the study on each data type (e.g., SNPs and gene expression data) respectively. To demonstrate the advantage of our proposed integrative approach, we compared the selected genes using the SRC method with the previously reported osteoporosis susceptive genes [5], [19]. To further validate the selected genes, we applied the method to the classification of osteoporosis patients with the selected gene expression and/or SNP data. Results showed that the SRC method is able to better locate genes significant for the diagnosis of osteoporosis patients than those from a single data set. In addition, our proposed SRC method gives better diagnosis results when compared with the T-test and Fisher-exact test. In particular, we identified two new osteoporosis risk genes (e.g., *‘DICER1’, ‘PTMA’*) through joint data analysis. These genes cannot be identified with single data set but show significant roles in osteoporosis etiology from studies published in existing literatures, which suggests that an integrated data analysis can lead to better identification of genes, resulting in improved diagnosis.

## Methods

In this section, we first describe the genomic data used in our study (Section 2.1). Then we present the SRC model (Sec. 2.2), the feature selection method (Sec. 2.3) and the SRC based gene/variable shaving algorithm (Sec. 2.4). Finally, we describe the method used for validating the selected genes (Sec. 2.5).

### 2.1 Data

We applied the SRC method to the integrative analysis of two data sets (i.e., gene expression data set and a SNP data set) from our osteoporosis study. We describe the data sets as follows.

The gene expression data were from female osteoporosis subjects with extremely low (N = 40) (cases) vs. high (N = 40) (controls) bone mineral densities (BMDs). In the present study we selected circulating monocytes as our target cells because circulating monocytes serve as progenitors of osteoclasts [21]–[23], and secrete osteoclastogenic cytokines, such as IL-1, IL-6, and TNF-α [24]–[26]. Circulating monocytes were isolated from 50 *ml* whole blood. After RNA extraction, expression levels of 22283 transcripts were quantified by Affymetrix Human Genome U133A 2.0 Array (Affymetrix, Santa Clara, CA). GCOS 1.2 (Gene Chip Operating Software) was used to process the probe-level raw data. We used the RMA (Robust Multi-array Average) algorithm [6] implemented in R package to transform the probe-level raw data into gene expression data.

The SNP data set was from osteoporosis vs. healthy subjects, which were recruited with the purpose of identifying genetic factors underlying osteoporosis via genome-wide association study in a total of 1000 random subjects (age: 50.3+18.3 years) [17]. These subjects were genotyped with Human Mapping 500K Array Set that examined about 500000 SNPs with a relatively even distribution across the entire human genome. Since the gene expression data currently used are from female samples, we first distributed the total 501 female samples according to the hip Z-score of BMD and then selected the bottom 100 and top 100 subjects of the BMD phenotypic distribution as cases and controls, respectively. A total of 376821 eligible SNPs were used in final analysis. In addition, we randomly selected 70 cases and 70 controls as training data for gene selection, and the rest 30 cases and 30 controls were used as an independent testing data set.

**Figure 1:**
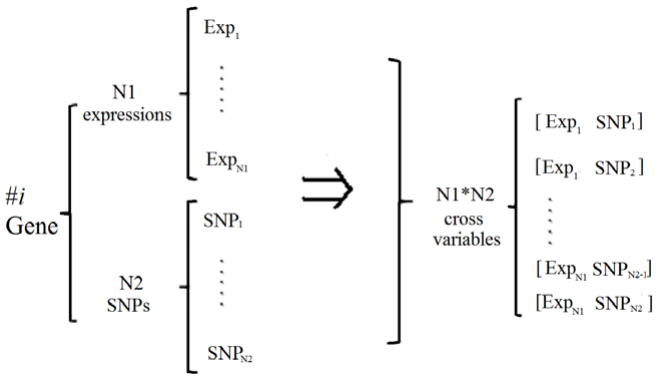
An illustration of the combination of two different types of data for the *i*th gene.

To perform joint data analysis, we generate a combined data set from the two single data sets, which are described as follows. For the *i*-th gene, there are 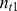 gene expressions and 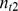 SNPs (in the gene expression data set, one gene may have one or more gene expressions; in the SNP data set, one gene usually has more than one SNPs). Thus for the *i*-th gene, we make a cross combination of the 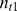 expressions and 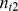 SNPs to obtain the 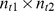 vector consisting of these two data (shown in Figure 1), which will be used for the selection of genes. Specifically, with the combination of gene expression (22283 expressions) and SNP data (376821 SNPs), we have a new data vector with 360930 variables (some of the genes do not appear in both data sets, which were not taken into consideration for the combined analysis). For each gene, the feature vector contains two sub-vectors: gene expression and SNP data, which will be used together as the input to our SRC method for joint data analysis.

### 2.2 SRC clustering algorithm

**Figure 2:**
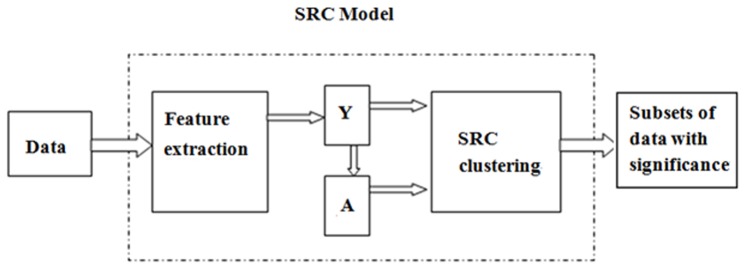
Diagram of SRC model for the data analysis using multi-features.

Figure 2 shows the diagram of the proposed SRC model. The ‘Feature extraction’ can be the output of a feature extraction method discussed above (*e.g.*, FSFS, CCA, GSVD), or statistical features such as the mean and variance. 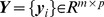 consists of feature vectors extracted from the data, where 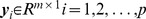 is the feature vector extracted for the *i*th gene/variable; *m* is the number of features (in this work, we employed m = 5 features for each gene in one type of data, as shown in Section 2.3 ‘Features selection’); and *p* is the number of gene/variables (for the gene expression data used in this work, p = 22283; for the SNP data, p = 376821). 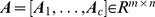 is the characteristic matrix that we will design to separate the data into *c* groups. For each group, 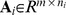 contains 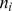 samples, and 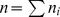. The ‘SRC clustering’ is to cluster each 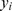 according to the characteristic matrix ***A*** that can be learned from the training data (the column number *n* is dependent on the number of features *m*. Detailed description can be seen in **Supporting Material S2**).

The description of the SRC algorithm is given in **Supporting Material S1**. The input of the SRC algorithm is the characteristic matrix ***A***, which is designed to cluster the data into different groups. In the previous studies, the characteristic matrix ***A*** was formed using the training samples [8]–[9]. In the current work, we design the characteristic matrix ***A*** with column vectors 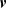 to designate different clusters with specific characteristics extracted from data. The clustering of features is based on both vector angle and amplitude (*e.g.*, measured using the L2 norm of the vector). The vector angle between the two column vectors 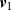 and 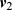 is defined by 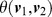, with 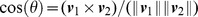. The design of characteristic matrix ***A***, is described in **Supporting Material S2** and the description of extracted feature vectors 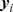 will be given in Section 2.3.

### 2.3 Features selection

As shown in Figure 2, we used the extracted features ***Y*** from the original data as the input for the variable/gene selection. In this work, we proposed to employ five features. Specifically, for each gene/variable (gene expressions or SNPs), we have a feature vector defined by Eq. (1):
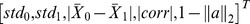
(1)where 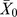 and 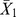, 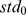 and 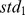 are the means and standard deviation of control and case group respectively; *corr* is the Pearson correlation coefficient between each gene expression (or SNP) data and the healthy status (‘1’ for patients, and ‘0’ for controls); and *a* is the normalized amplitude of vector 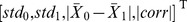 Features 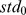 and 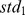 reflect the difference within each group, mean difference 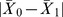 and the Pearson correlation coefficient 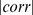 reflect the difference between the control and case groups. Therefore, feature vectors with smaller first two entries while larger last three entries are considered to be significant for discriminating control and case groups.

For a data set, we can extract feature vectors for each variable/gene to construct a feature matrix 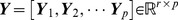 where *p* is the number of variables/genes, and *r* is the number of features. In this work, r = 5 for the analysis of one type of data and r = 10 for the integrative analysis of two types of data. For the integrative analysis, since each gene has two types of data, features were extracted from both sub-vectors as illustrated in Figure 1. Since different feature has different range, a scale transformation is performed for each row of the feature matrix ***Y*** so that all the entries of ***Y*** are within the range of [0, 1]. In **Supporting Material S3**, we discuss the significance of these selected features and the relationships between them (see Fig. 1 and Fig. 2 in **Supporting Material S3**).

### 2.4 The SRC based gene/variable shaving

**Figure 3:**
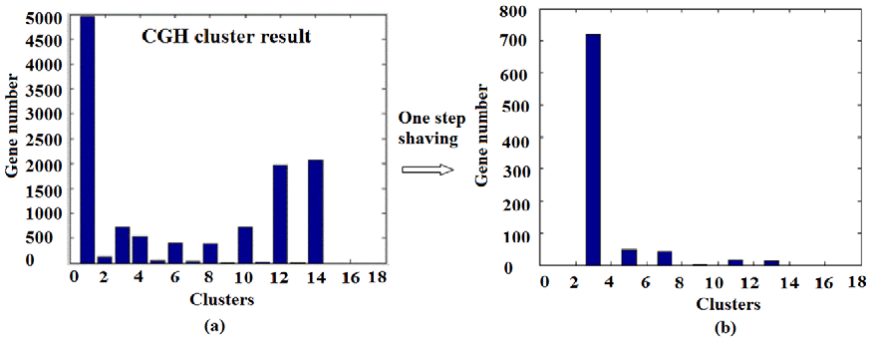
Diagram of gene shaving by SRC. (a) all genes were clustered into different groups; and (b) only clusters of a particular significance were selected for further analysis.

Once we have the characteristic matrix ***A*** and the feature vectors ***Y***, the SRC algorithm given by **Supporting Material S1** can be applied for gene shaving or for the selection of significant genes. Figure 3 gives an illustration of the gene/variable shaving process using the SRC based method. As shown in Figure 3, all genes were first grouped into different clusters using the SRC algorithm (Figure 3 (a)). Since each group is designed to have different statistical significances, those genes that fall into the group(s) of a particular significance can be selected for further analysis (Figure 3 (b)), while others will be shaved off. The process will continue until the number of remaining genes meets the threshold set with prior knowledge.

**Figure 4:**
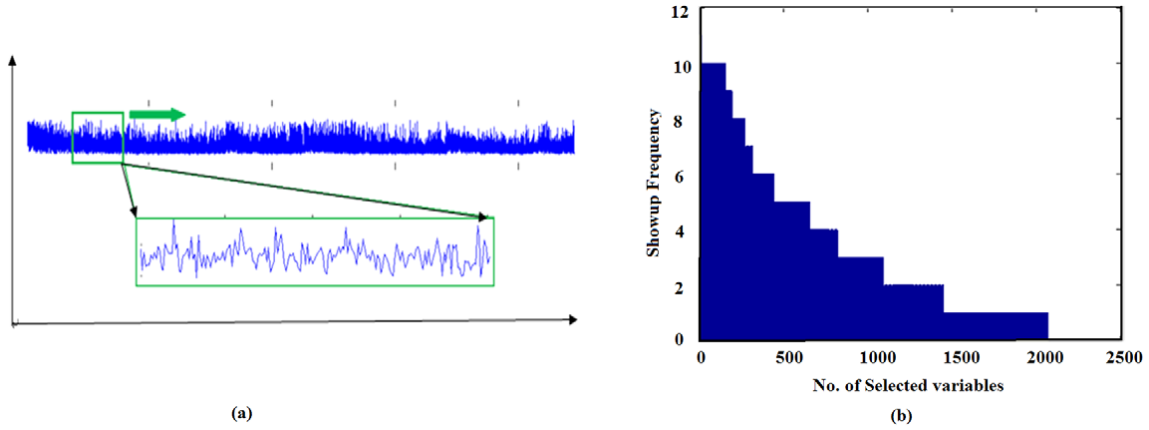
The SRC based gene shaving with a sliding window. (a) Gene selection was performed within each sliding window; (b) Genes selected with Fisher-Yates Shuffling algorithm; the higher the selected frequencies, the more significance of the variable (see the explanation of Algorithm 2).

When the data set is very large, which is always the case for genomic data, a sliding window is applied and the gene selection is performed within the window (Figure 4 (a)) in order to account for local variations in the data. We also performed data shuffle with Fisher-Yates Shuffling algorithm [20] to reduce bias. Those genes selected with highest frequencies will be the ones that are most significant, as shown in Figure 4 (b). A description of the SRC based gene shaving algorithm with a sliding window is given as ***Algorithm 2***.

**Algorithm 2: SRC based gene shaving algorithm.**

1. Set the window length, the window sliding step length, and the starting point;
2. For the *l*-th iteration, perform gene selection within a window and record the selected genes;
3. Slide the window from the starting point with the pre-set step length, and repeat Step 2 until the window reaches the ending point of the data sample.
4. Shuffle the data with Fisher-Yates Shuffling algorithm; and repeat Step 2–Step 3.
5. Compare the gene list generated by all *l* iterations with that generated by previous *l*-1 iterations; if the gene list percentage similarity (PS) is higher than a pre-set threshold, exit; otherwise, go to Step 4.

In ***Step*** 5, the gene list percentage similarity (*PS*) between the two different lists is defined by Eq. (2).
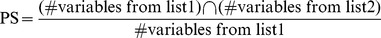
(2)


### 2.5 Validation

Two strategies were taken to validate our method: 1.We test if the osteoporosis susceptive genes selected by our method can be confirmed with previously reported ones, in addition to the identification of new genes. 2. We test if gene expression/SNPs corresponding to selected genes are able to identify osteoporosis patients from healthy controls, which are quantified with classification ratio (CR). We define CR in this work as the ratio between the number of correctly classified samples and total number of samples. We conducted the leave-one-out (LOO) cross validation. We compared our method with current ones for gene selection (e.g., T-test for expression data and Fisher-exact test for SNP data). In addition, we compared the results of using combined data sets (gene expression and SNPs) with those of using each individual data set, in order to demonstrate the advantage of the proposed integration approach.

## Results

One goal of our work was to study whether integrative analysis approach with our proposed SRC algorithm can lead to better identification of susceptible genes for the diagnosis of complex diseases such as osteoporosis. We conducted the analysis on two data sets with different structures, ranges and formats: gene expression (40 patients/40 controls, 22283 gene expressions), and SNP association analysis data (70 patients/70 controls, 376821 SNPs) from osteoporosis study. To validate the selected genes, we compared our selected gene lists with those previously reported. In addition, we tested if the selected genes can result in better diagnosis of osteoporosis. We performed the leave-one-out (LOO) cross validation for both data sets and the test using an independent SNP data set (30 patients/30 controls, 376821 SNPs). To demonstrate the performance of our SRC method, the results of our SRC method for gene selection were compared with those from both T-test and Fisher-exact test. In addition, the results of classifying osteoporosis using individual and joint data sets were compared, showing that integrated analysis can give higher diagnosis accuracy.

### 3.1 Comparison of selected genes using different methods

**Figure 5:**
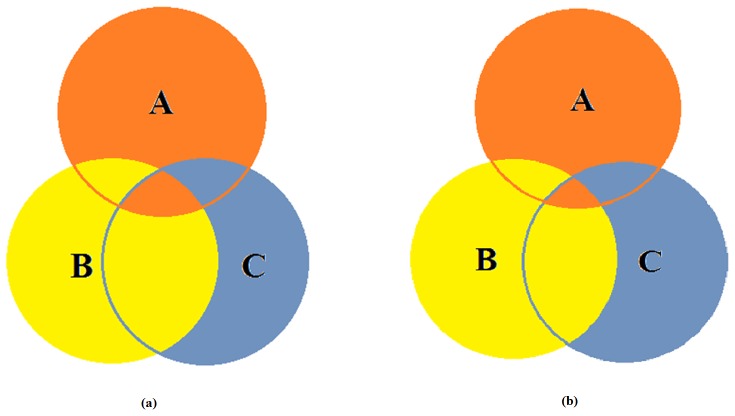
Comparison of the selected variables (expressions/SNPs) using the Venn diagram. (a) comparison of the first 500 gene expressions selected with integrative analysis by the SRC method (orange area A), individual data analysis using SRC method (yellow area B) and using T-test (blue area C) respectively; (b) comparison of the first 1000 SNPs selected with integrative analysis with the SRC method (orange area A), individual analysis with the SRC method (yellow area B) and Fisher- exact test (blue area C) respectively. **Supporting Materials S4 and S5** give the first 50 variables (gene expressions/SNPs) selected using individual analysis. **Supporting Material S6** gives the first 50 variables (gene expressions and SNPs) selected using integrative analysis, and Fig. 1 in Supporting Material S6 compares the selected variables in cases and controls.

To show the differences between integrative analysis and individual analysis using both the SRC and conventional feature selection methods (e.g., T-test and Fisher-exact test), we compared the first 500 gene expressions and 1000 SNPs selected by different methods using the Venn diagram, as shown in Figure 5. The intersection between individual analysis using the SRC method and T-test for the gene expression data is about 45% (Figure 5 (a), the intersection between B and C); the intersection between the SRC method and Fisher-exact test for individual analysis of SNPs is about 39% (Figure 5 (b), the intersection between B and C area); and the intersection between combined analysis using SRC method and individual analysis is below 10% (A and B, A and C area for both Figure 5 (a) and (b)).

When compared the results with the previous study, our SRC based variable selection method was able to locate osteoporosis susceptive genes that were reported before [19] such as *‘ESRRA’, ‘CALM1’, ‘CALM1’, ‘SPARC’,‘LRP1’*, *‘THSD4’, ‘CRHR1’, ‘HSD11B1’, ‘THSD7A’, ‘BMPR1B’, ‘ADCY10’, ‘PRL’, ‘CA8’*, *et. al.* **Supporting Material S6** gives the first 50 gene expressions and SNPs selected using joint data sets. In particular, there were some significant genes that were not identified by individual data analysis, such as *‘DICER1’, ‘PTMA’ etc.* However, evidences have existed to show that these genes may be associated with the osteoporosis disease (details in the Section of Discussion).

### 3.2 Validation of the selected genes on the diagnosis of osteoporosis

We further validated the selected genes for the diagnosis of osteoporosis subjects, whose accuracy was measured with the LOO cross-validation.

**Figure 6:**
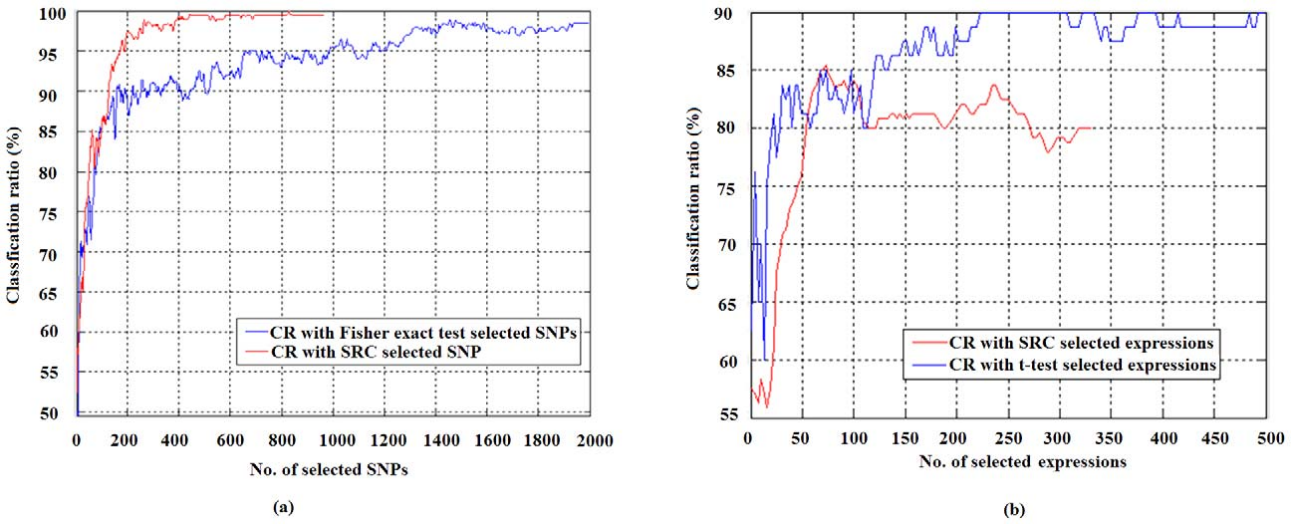
Comparison of classification accuracy with LOO cross-validation using the selected variables. (a)Validation results for the gene expression data by SRC the method and t-test method respectively. (b)Validation results for the SNP data by the SRC method and Fisher-exact test method respectively.

First, we showed that using gene expression or SNPs selected with our proposed SRC method can give higher diagnosis accuracy than that of the current methods such as the t-test and Fisher exact test. When using selected gene expression data alone to identify the osteoporosis patients, we got the highest classification ratio (CR) (86.25%) with 73 expression data, while for t-test method we got the highest CR of 90% with 225 gene expressions, as shown in Figure 6 (a). For the SNP data set, we got the highest CR (100%) with 883 SNPs using the SRC, while the highest CR (96.5%) with 1460 SNPs using Fisher-exact test, as shown in Figure 6 (b). Both results indicate that the classification of osteoporosis with the SRC is significantly better than the t-test and Fisher-exact test, which are currently widely used for the study of osteoporosis diseases.

**Figure 7:**
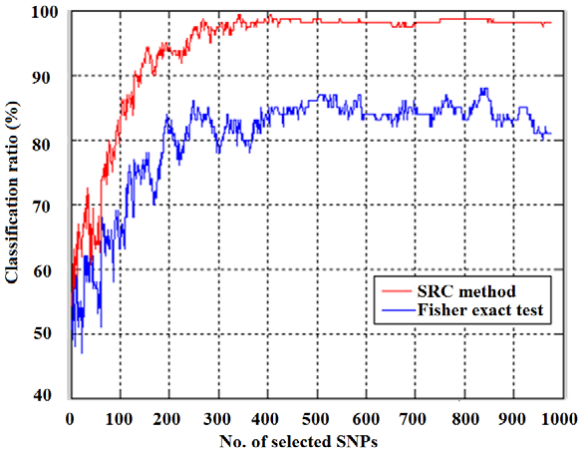
The classification of osteoporosis patients on an independent SNP data set.

Besides the LOO cross-validation, we also performed a blind test on the selection of OP susceptive genes. We applied the method to the classification of osteoporosis on an independent SNP data set (30 patients/30 controls, 376821 SNPs), and compared the results with the Fisher-exact test analysis, as shown in Figure 7. Using the SRC selected SNPs, the classification ratio reached as high as 98.33%, while using the SNPs selected with Fisher-exact test, the highest CR was only 88.33%, as shown in Figure 7.

**Figure 8:**
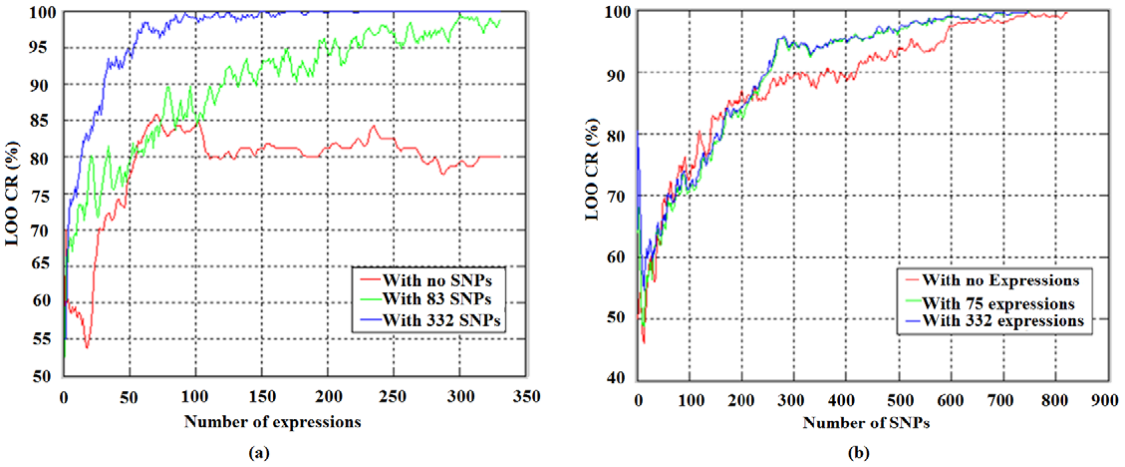
Using selected variables from both data sets for the classification of osteoporosis patients. (a) Classification accuracy using gene expressions along with N = 0, 83, 332 selected SNPs employed for the classification. (b) Classification accuracy using SNPs along with N = 0, 75, 332 selected gene expressions employed for the classification.

Finally, we compared the classification accuracy of using combined data set with that of using individual data. For the combined data set, each selected feature vector contains two sub-vectors (SNP and gene expression sub-vector). Therefore, we calculated the CRs of using the whole feature vector and each sub-vector respectively. Figure 8 demonstrates that higher identification accuracy can be obtained with complementary information from both data sets than using an individual data.

## Discussion

Identification of candidate genes from vast amount of genomic data for the diagnosis of complex disease has been a significant challenge. In this study, we address the problem by developing a sparse representation based clustering (SRC) method that can be used for integrative analyses of various types of genomic data. We applied the SRC based gene/variable selection method to the identification of genes associated with osteoporosis diseases. The SRC method demonstrates two advantages: 1. Different from other analysis methods, the SRC method employs multi-features extracted from diverse data sets rather than the original raw data, facilitating the integration of data with different formats and structures. 2. The SRC method outperforms several currently used significance test methods such as the T-test and Fisher-exact test, by employing a more sophisticated clustering based approach.

When compared with previously reported osteoporosis susceptible genes, the SRC based gene shaving method not only identified genes that were previously reported [19], such as *‘THSD4’, ‘CRHR1’, ‘HSD11B1’, ‘THSD7A’, ‘BMPR1B’ ‘ADCY10’, ‘PRL’, ‘CA8’, ‘ESRRA’, ‘CALM1’, ‘CALM1’, ‘SPARC’, ‘LRP1’*, but also new susceptive genes (*‘DICER1’, ‘PTMA’ et. al.*). Evidences [27]–[35] have shown that these genes play a significant role in the etiology of osteoporosis, as discussed below. In particular, it should be noticed that these genes cannot be identified with the analysis of an individual data set, demonstrating the advantage of integrative analysis of multiple types of data. In the following, we further elucidate the relevance of the identified new genes to osteoporosis from previous studies.

*DICER1* (dicer 1, ribonuclease type III), also known as Dicer, is essential for microRNA (miRNA) processing and the synthesis of small interfering RNAs from long double-stranded RNA [27]. This gene is located at 14q32.13. Some evidences suggested that *DICER1* was closely associated with bone metabolism. For example, Dicer in osteoclasts controls activity of bone resorption in vivo [28]. Gene silencing of Dicer by small interfering RNA revealed global inhibition of osteoclast transcription factor expression and function, decreased osteoclastogenesis, and decreased bone resorption in vitro [29]. Dicer possessed significantly decreased miR-21 levels and increased PDCD4 protein levels so that RANKL-induced osteoclastogenesis was impaired in those cells [30]. Dicer generated miRs are necessary for two periods of bone formation, to promote osteoblast differentiation before birth, and control bone accrual in the adult [31].

*PTMA* (prothymosin, alpha) may play important roles in osteoporosis. Over-expressed PTMA enhanced p53 transcriptional activity in reporter gene assays for p53 target gene promoters hdm2, p21, and cyclin G, and increased mRNA and protein levels for endogenous p53 target genes, hdm2 and p21 [32]. Some studies reported that p53 regulates osteoblast differentiation, bone formation, and osteoblast-dependent osteoclast differentiation [33]. As p53+/m mice age, they reveal an early onset of phenotypes associated with aging. A recent serendipitously also generated p53 mutant allele resulted in a hypermorphic version of p53 that mediates decreased longevity. The reduced longevity is accompanied by the accelerated onset of a variety of aging phenotypes. These include reduced longevity, osteoporosis, generalized organ atrophy and a diminished stress tolerance [34], [35].

When we compared the selected gene list with that selected by a t-test and Fisher-exact test (see Figure 5(a) and (b)), it can be seen that the variables (SNPs/expressions) selected by the SRC method are quite different (>50% in the number). However, the integrative analysis with the SRC method selects two sub-vectors simultaneously and gives better classification accuracy, because of the use of complementary information. For example, using the SNP data, the SRC based method can give the highest CR of 100% than 97.14% of using Fisher exact test with less number of SNPs (see Figure 6). When using both types of data for the cross validation, the CR of using combined data sets with the SRC method is much higher than that of using one type of data (see Figure 8), demonstrating the significance of integrative data analysis. In addition, when performing a blind test on an independent SNP data set (30 cases 30 controls), the CR can be as high as 98.33% with the SRC method; with Fisher-exact test selected SNPs, the highest classification ratio is only 88.33%, showing the advantage of the SRC method.

In our integrative analysis method, gene expression and SNP data were combined in terms of each gene. Therefore, our method uses joint information from two complementary data rather than from a single type of data, which can lead to the increase of reliability in gene identification. Besides the significance discussed above, the integrative analysis employed in this work can be generalized to include more than two types of data. We are currently testing the method for the integration of multiple genomic data from the TCGA database for improved diagnosis of cancers such as the leukemia. In addition, the sample size in this work is small (100/100 cases/controls for the SNP data set and 40/40 subjects for the gene expression data set). To further validate the proposed method and the significance of the selected genes, larger data sets will be tested.

## Supporting Information

Supporting Material S1 **Sparse Representation-based clustering (SRC) algorithm.**
(DOCX)

Supporting Material S2 **Design of Characteristic matrix** ***A*** **.**
(DOCX)

Supporting Material S3 **Significance of the selected features.**
(DOCX)

Supporting Material S4 **The first 50 selected expressions (with corresponding gene names) by the individual analysis of OP gene expression data.**
(DOCX)

Supporting Material S5 **The first 50 selected SNPs (with corresponding gene names) by the individual analysis of OP SNP data.**
(DOCX)

Supporting Material S6 **The first 50 selected SNPs and gene expressions selected (with corresponding gene names) by the joint analysis of both OP SNP and gene expression data.**
(DOCX)

## References

[1] Yang HH, Liu JY, Sui J, Pearlson Gand Calhoun VD (2010) A hybrid machine learning method for fusing fMRI and genetic data: combining both improves classification of schizophrenia. Frontiers in Human Neuroscience 4: 1–9. 21119772 10.3389/fnhum.2010.00192 PMC2990459

[2] Soneson C, Lilljebjörn H, Fioretos T, Fontes M (2010) Integrative analysis of gene expression and copy number alterations using canonical correlation analysis. BMC Bioinformatics 11: 191. 20398334 10.1186/1471-2105-11-191 PMC2873536

[3] Cao KAL, Martin PG, Robert-Granie C, Besse P (2009) Sparse canonical methods for biological data integration: application to a cross platform study. BMC Bioinformatics 10: 34. 19171069 10.1186/1471-2105-10-34 PMC2640358

[4] Berger JA, Hautaniemi S, Mitra SK, Astola J (2006) Jointly Analyzing Genes Expression and Copy Number Data in Breast Cancer using Data Reduction model. IEEE T Comput B I 3 (1) 2–16. 10.1109/TCBB.2006.10 17048389

[5] Liu YJ, Shen H, Xiao P, Xiong DH, Li LH, et al (2005) Molecular Genetic Studies of Gene Identification for Osteoporosis: A 2004 Update. Journal of Bone and Mineral Research 21 (10) 1551–1535. 10.1359/JBMR.051002 PMC1829484 16995806

[6] Wang P, Kim Y, Pollack J, Narasimhan B, Tibshirani R (2005) A Method for Calling Gains and Losses in Array CGH Data. Biostatistics 6: 45–58. 15618527 10.1093/biostatistics/kxh017

[7] Hautaniemi S, Ringner M, Kauraniemi P, Autio R, Edgren H, et al (2004) A Strategy for Identifying Putative Causes of Gene Expression Variation in Human Cancers. J Franklin Inst 341: 77–88.

[8] Loo LWM, Grove DI, Williams EM, Neal CL, Cousens LA, et al (2004) Array Comparative Genomic Hybridization Analysis of Genomic Alterations in Breast Cancer Subtypes. Cancer Research 64: 8541–8549. 15574760 10.1158/0008-5472.CAN-04-1992

[9] Cao HB, Deng HW, Li M, Wang YP (2012) Classification of Multicolor Fluorescence In-situ Hybridization (M-FISH) Images with Sparse Representation. IEEE Tans Nano Biosciences 11: 111–118. 10.1109/TNB.2012.2189414 PMC4165853 22665392

[10] Melton LJ, Chrischilles EA, Cooper C, Lane AW, Riggs BL (2005) How many women have osteoporosis? JBMR Anniversary Classic. J Bone Miner Res 20: 886–892. 15931736 10.1359/jbmr.2005.20.5.886

[11] Cummings SR, Nevitt MC, Browner WS, Stone K, Fox KM, et al (1995) Risk factors for hip fracture in white women. Study of Osteoporotic Fractures Research Group. N Engl J Med 332: 767–773. 7862179 10.1056/NEJM199503233321202

[12] Deng HW, Mahaney MC, Williams JT, Li J, Conway T, et al (2002) Relevance of the genes for bone mass variation to susceptibility to osteoporotic fractures and its implications to gene search for complex human diseases. Genet Epidemiol 22: 12–25. 11754470 10.1002/gepi.1040

[13] Liu PY, Qin YJ, Recker RR, Deng HW (2004) Evidence for a major gene underlying bone size variation in the Chinese. Am J Hum Biol 16: 68–77. 14689517 10.1002/ajhb.10240

[14] Jian WX, Long JR, Li MX, Liu XH, Deng HW (2005) Genetic determination of variation and covariation of bone mineral density at the hip and spine in a Chinese population. J Bone Miner Metab 23: 181–185. 15750698 10.1007/s00774-004-0558-3

[15] Hsu YH, Zillikens MC, Wilson SG, Farber CR, Demissie S, et al (2010) An integration of genome-wide association study and gene expression profiling to prioritize the discovery of novel susceptibility Loci for osteoporosis-related traits. PLoS Genet 6 (6) 1–16. 10.1371/journal.pgen.1000977 PMC2883588 20548944

[16] Deng FY, Lei SF, Chen XD, Tan LJ, Zhu XZ, et al (2011) An integrative study ascertained SOD2 as a susceptibility gene for osteoporosis in Chinese. J Bone Miner Res 26 (11) 2695–2701. 21773993 10.1002/jbmr.471 PMC3375319

[17] Xiong DH, Liu XG, Guo YF, Tan LJ, Wang L, et al (2009) Genome-wide association and follow-up replication studies identified ADAMTS18 and TGFBR3 as bone mass candidate genes in different ethnic groups. Am J Hum Genet 84 (3) 388–398. 19249006 10.1016/j.ajhg.2009.01.025 PMC2667986

[18] Yang TL, Chen XD, Guo Y, Lei SF, Wang JT, et al (2008) Genome-wide copy-number-variation study identified a susceptibility gene,UGT2B17, for osteoporosis. Am J Hum Genet 83 (6) 663–674. 18992858 10.1016/j.ajhg.2008.10.006 PMC2667994

[19] Xu XH, Dong SS, Guo Y, Yang TL, Lei SF, et al (2010) Molecular Genetic Studies of Gene Identification for Osteoporosis: The 2009 Update,. Endocr Rev 31: 447–505. 20357209 10.1210/er.2009-0032 PMC3365849

[20] Fisher RA, Yates F (1948) Statistical tables for biological, agricultural and medical research, 3rd ed. London: Oliver and Boyd. pp. 26–27.

[21] Udagawa N, Takahashi N, Akatsu T, Tanaka H, Sasaki T, et al (1990) Origin of osteoclasts: mature monocytes and macrophages are capable of differentiating into osteoclasts under a suitable microenvironment prepared by bone marrow-derived stromal cells. Proc Natl Acad Sci U S A 87 (18) 7260–7264. 2169622 10.1073/pnas.87.18.7260 PMC54723

[22] Zambonin ZA, Teti A, Primavera MV (1984) Monocytes from circulating blood fuse in vitro with purified osteoclasts in primary culture. J Cell Sci 66: 335–342. 6378944 10.1242/jcs.66.1.335

[23] Fujikawa Y, Quinn JM, Sabokbar A, McGee J, Athanasou NA (1996) The human osteoclast precursor circulates in the monocyte fraction. Endocrinology 137 (9) 4058–4060. 8756585 10.1210/endo.137.9.8756585

[24] Cohen-Solal ME, Graulet AM, Denne MA, Gueris J, Baylink D, et al (1993) Peripheral monocyte culture suppernatants of menopausal women can induce bone resorption: involvement of cytokines. J Clin Endocrinol Metab 77 (6) 1648–1653. 8263153 10.1210/jcem.77.6.8263153

[25] Eghbali-Fatourechi G, Khosla S, Sanyal A, Boyle WJ, Lacey DL, et al (2003) Role of RANK ligand in mediating increased bone resorption in early postmenopausal women. J Clin Invest 111 (8) 1221–1230. 12697741 10.1172/JCI17215 PMC152939

[26] Pacifici R (1996) Estrogen, cytokines, and pathogenesis of postmenopausal osteoporosis. J Bone Miner Res 11 (8) 1043–1051. 8854239 10.1002/jbmr.5650110802

[27] Nagaraja AK, Andreu-Vieyra C, Franco HL, Ma L, Chen R, et al (2008) Deletion of Dicer in somatic cells of the female reproductive tract causes sterility. Mol Endocrinol 22 (10) 2336–2352. 18687735 10.1210/me.2008-0142 PMC2582529

[28] Mizoguchi F, Izu Y, Hayata T, Hemmi H, Nakashima K, et al (2010) Osteoclast-specific Dicer gene deficiency suppresses osteoclastic bone resorption. J Cell Biochem 109 (5) 866–875. 20039311 10.1002/jcb.22228

[29] Sugatani T, Hruska KA (2009) Impaired micro-RNA pathways diminish osteoclast differentiation and function. J Biol Chem 284 (7) 4667–4678. 19059913 10.1074/jbc.M805777200 PMC2640963

[30] Sugatani T, Vacher J, Hruska KA (2011) A microRNA expression signature of osteoclastogenesis. Blood 117 (13) 3648–3657. 21273303 10.1182/blood-2010-10-311415 PMC3072882

[31] Gaur T, Hussain S, Mudhasani R, Parulkar I, Colby JL, et al (2010) Dicer inactivation in osteoprogenitor cells compromises fetal survival and bone formation, while excision in differentiated osteoblasts increases bone mass in the adult mouse. Dev Biol 340 (1) 10–21. 20079730 10.1016/j.ydbio.2010.01.008 PMC2840721

[32] Kobayashi T, Wang T, Maezawa M, Kobayashi M, Ohnishi S, et al (2006) Over expression of the oncoprotein prothymosin alpha triggers a p53 response that involves p53 acetylation. Cancer Res 66 (6) 3137–3144. 16540664 10.1158/0008-5472.CAN-05-2112

[33] Wang X, Kua HY, Hu Y, Guo K, Zeng Q, et al (2006) p53 functions as a negative regulator of osteoblastogenesis, osteoblast-dependent osteoclastogenesis, and bone remodeling. J Cell Biol 172 (1) 115–125. 16380437 10.1083/jcb.200507106 PMC2063539

[34] Dumble M, Gatza C, Tyner S, Venkatachalam S, Donehower LA (2004) Insights into aging obtained from p53 mutant mouse models. Ann N Y Acad Sci 1019: 171–177. 15247009 10.1196/annals.1297.027

[35] Tyner SD, Venkatachalam S, Choi J, Jones S, Ghebraniousk N, et al (2002) p53 mutant mice that display early ageing-associated phenotypes. Nature 415 (6867) 45–53. 11780111 10.1038/415045a

