# Supplementary material for: Identification of Genes for Complex Diseases Using Integrated Analysis of Multiple Types of Genomic Data
Source: PLoS One. 2012 Sep 5;7(9):e42755. doi: 10.1371/journal.pone.0042755 (PMC3434191; doi:10.1371/journal.pone.0042755)
Supplement: Supporting Material S1 — Sparse Representation-based clustering (SRC) algorithm. (DOCX) [file pone.0042755.s001.docx]

**Supporting Materials 1: Sparse Representation-based clustering (SRC) algorithm**

The ‘SRC clustering’ algorithm is based on the solution of the L1-minimization problem (P1):

(P1) $\hat{\boldsymbol{x}}=argmin\left\| \boldsymbol{x} \right\|_{1}$ subject to $\boldsymbol{Ax}\boldsymbol{=}\boldsymbol{y}$, (1)

Where $\boldsymbol{A}\in R^{m\times n}$, $\boldsymbol{x}\in R^{n\times1}$, $\boldsymbol{y}\in R^{m\times1}$, and $m\ll n$*.*The characteristic matrix $\boldsymbol{A}$is designed to cluster the data into $c$different groups. When $\boldsymbol{y}$ belongs to one of the groups, the sparse solution $\boldsymbol{x}$ of the problem (P1) has a few non-zeros entries concentrating on a particular set of locations, corresponding to that group.

***Algorithm 1: Sparse Representation-based clustering (SRC) algorithm:***

1. Inputs: characteristic matrix $\boldsymbol{A}=[\boldsymbol{A}_{1},\ldots,\boldsymbol{A}_{c}]\boldsymbol{\in}R^{m\times n}$and vectors$\boldsymbol{Y}\boldsymbol{=}\left\{ \boldsymbol{y}_{i} \right\}\boldsymbol{\in}R^{m\times p};$
2. Normalize the columns of $\boldsymbol{A}$ to have unit L2-norm;
3. Solve the L1-norm minimization problem (P1) defined by Equation (1), with $\boldsymbol{A}$ and $\boldsymbol{y}_{i}$ as input;
4. Calculate the vector angle$\theta_{k}\left( \boldsymbol{y}_{i},{\boldsymbol{A}\delta}_{k}(\boldsymbol{x}) \right)$, $k\in\{1, 2, \ldots,c\}$;
5. $Identity\left( \boldsymbol{y}_{i} \right)=arg \min_{k}( \theta_{k})$;

In the algorithm described above,$\delta_{k}(\boldsymbol{x})\neq0$for the entries corresponding to the $k$-th group; $\delta_{k}(\boldsymbol{x})$ =$\boldsymbol{0}$for the$j$-th group corresponded entries where $j\neq k$; ${{\hat{\boldsymbol{y}}}_{k}\boldsymbol{=A}\delta}_{k}(\boldsymbol{x})$ is the approximation of $\boldsymbol{y}$ with vectors from the $k$-th group, $k\in\{1,2,\ldots,c\}$, and $\boldsymbol{y=}\sum_{k=1}^{c} {\hat{\boldsymbol{y}}}_{k}$**.** For Step 3, there are many algorithms for solving the L1-norm minimization problem (P1) [1][2]. In this work, we employed the Homotopy method that was originally proposed by Osborne et al. Donoho et al. proved that the Homotopy method runs much more rapidly than general-purpose linear programs (LP) solvers when sufficient sparsity is present. Specifically, if the underlying solution has $k$ nonzeros entries, it can be obtained with only $k$ iterative steps [1].

1. DonohoDAndTsaig Y, (2006). Fast solution of L1-norm minimization problems when the solution may be sparse. preprint, http://www.stanford.edu/ tsaig/research.html.
2. OsborneMR,PresnellB, and Turlach, BA, (2000) A new approach to variable selection in least squares problems. IMA J. Numerical Analysis 20:389–403.
