# Supplementary material for: Identification of Genes for Complex Diseases Using Integrated Analysis of Multiple Types of Genomic Data
Source: PLoS One. 2012 Sep 5;7(9):e42755. doi: 10.1371/journal.pone.0042755 (PMC3434191; doi:10.1371/journal.pone.0042755)
Supplement: Supporting Material S2 — Design of Characteristic matrix A . (DOCX) [file pone.0042755.s002.docx]

**Supporting Materials 2 Design of Characteristic matrix** $\mathbf{A}$

If *m* number of features is used for clustering, there will be$c=2^{m}-1$ possible groups. We now discuss the design of characteristic matrix $\boldsymbol{A=}\{\boldsymbol{A}_{k}\}$, $k=1,\ldots,c,$to separate these *c* classes. We designate each group with a central column vector $\boldsymbol{V}_{k}\boldsymbol{\in}R^{m\times1}$ of binary value, with different 1 and 0 elements. Then we design the characteristic matrix of the $k$-th group$\boldsymbol{A}_{k}=\{\boldsymbol{V}_{k_{i}}\}\boldsymbol{\in}R^{m\times n_{i}}$, where $i=1,\ldots,n_{i}$, and $n_{i}>m$; $\boldsymbol{V}_{k_{i}}=\boldsymbol{V}_{k}+\boldsymbol{V}_{0}$, and $\boldsymbol{V}_{0}$ is a random vector with small amplitude. The relation between $\boldsymbol{V}_{k_{i}}$ and $\boldsymbol{V}_{k}$ is given by Eq. (2) to guarantee that different cluster groups designated by the corresponding vector $\boldsymbol{V}_{k}$ are separated

$\theta\left( \boldsymbol{V}_{k_{i}},\boldsymbol{V}_{k} \right)<1/2\min_{k\neq j} \theta\left( \boldsymbol{V}_{k},\boldsymbol{V}_{j} \right)$ (1)

It is also natural to require that

$rank\left( \boldsymbol{A}_{k} \right)=m$, (2)

$w$here $k=1,\ldots,c$.

In addition to the above requirements, a valid $\boldsymbol{v}$for the SRC based classifier should give a sparse solution $\boldsymbol{x}$ whose nonzero entries concentrate mostly on one group, rather than spread evenly over all groups. To be more specific, we use the Sparsity Concentration Index (SCI) proposed in^17^to measure how concentrated the feature vectors are on a single group in the data:

$SCI\left( \boldsymbol{x} \right)=\frac{c*\max_{i} \frac{\left\| \boldsymbol{\delta}_{i}\left( \boldsymbol{x} \right) \right\|_{1}}{\left\| \boldsymbol{x} \right\|_{1}}-1}{c-1}\in[0,1]$ (3)

where$c$ is the number of groups. For a solution $\hat{\boldsymbol{x}}$ found by the SRC algorithm, if $SCI\left( \hat{\boldsymbol{x}} \right)=1,$the feature vector $\mathbf{y}$ is represented using only vectors from a particular group; if $SCI\left( \hat{\boldsymbol{x}} \right)=0$, the sparse coefficients are spread evenly over all groups. We choose a threshold $\tau\in[0,1]$ and accept a$\boldsymbol{v}$as valid if $SCI\left( \hat{x} \right)>\tau$; otherwise, reject it as invalid.
