# Supplementary material for: Identification of Genes for Complex Diseases Using Integrated Analysis of Multiple Types of Genomic Data
Source: PLoS One. 2012 Sep 5;7(9):e42755. doi: 10.1371/journal.pone.0042755 (PMC3434191; doi:10.1371/journal.pone.0042755)
Supplement: Supporting Material S3 — Significance of the selected features. (DOCX) [file pone.0042755.s003.docx]

**Supporting Materials 3: Significance of the selected features**

It has been proven that the Pearson correlation coefficient $|corr|$and p-values from ANOVA test are closely related [1]. It can be shown that the p-values for the t-test decreases monotonically with the increase of the Pearson correlation coefficient $|corr|$, as illustrated inFigure 1 (a). In Figure 1 (b) we can see that the p-values from Fisher-exact test, commonly used SNP association test, decides the range of$|corr|$. Fisher-exact test does not consider the variances within in a group, while $|corr|$ employed in the SRC method requires small intro-group variance.


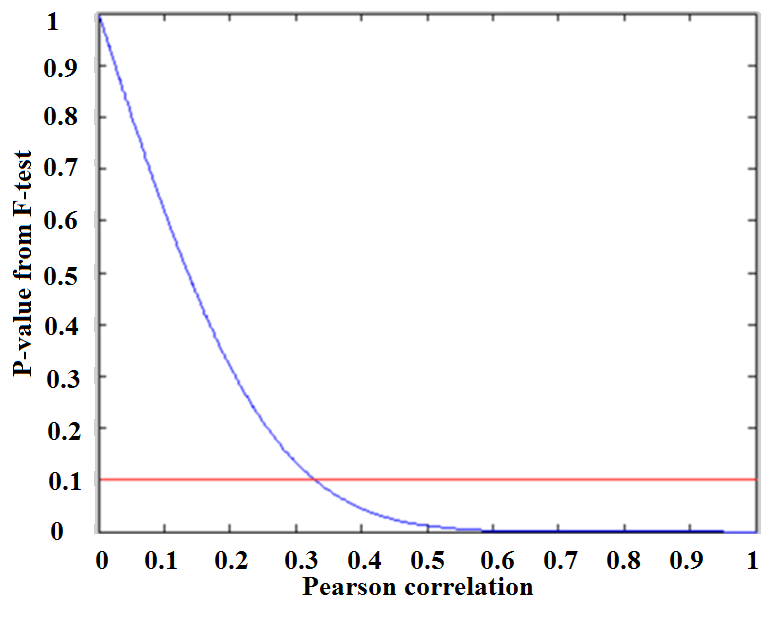

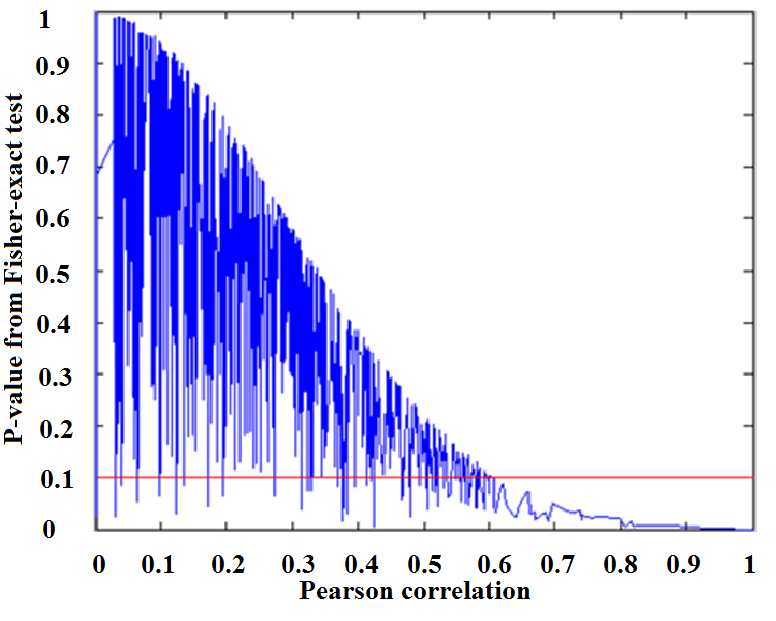


(a) (b)

**Figure 1 Validation of Pearson correlation coefficient** $\boldsymbol{|corr|}$**.** (a) Relationship between P-value from t-test and Pearson correlation coefficient $|corr|$; (b) Relationship between P-value from Fisher-exact test and Pearson correlation coefficient $|corr|$

When comparing the difference of two groups, Pearson correlation coefficient $|corr|$takes into both the means and the standard deviations into consideration. As shown in Figure 2 (a), the Person correlation and the absolute mean differences of two groups have strong correlations. However, variables with big absolute mean differences are significant for the identification of two different groups. Thus, it is necessary to take the absolute mean differences of the two groups as one of the features.


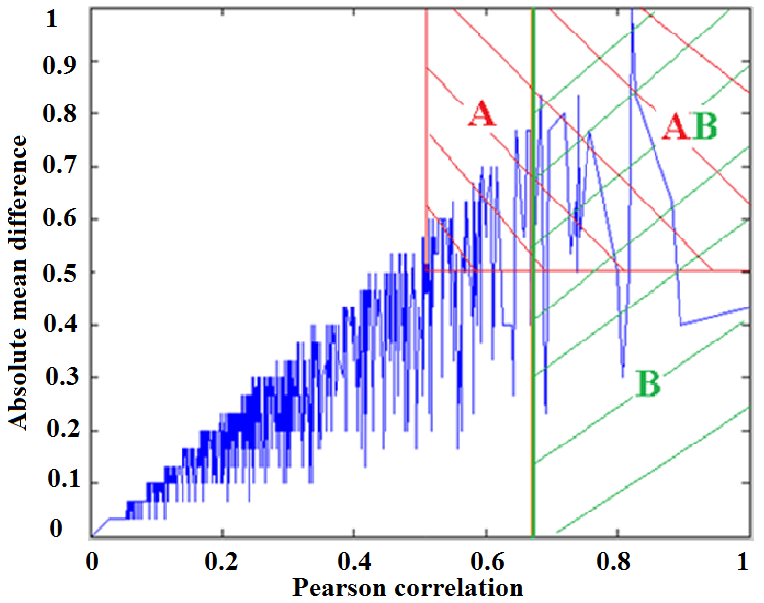

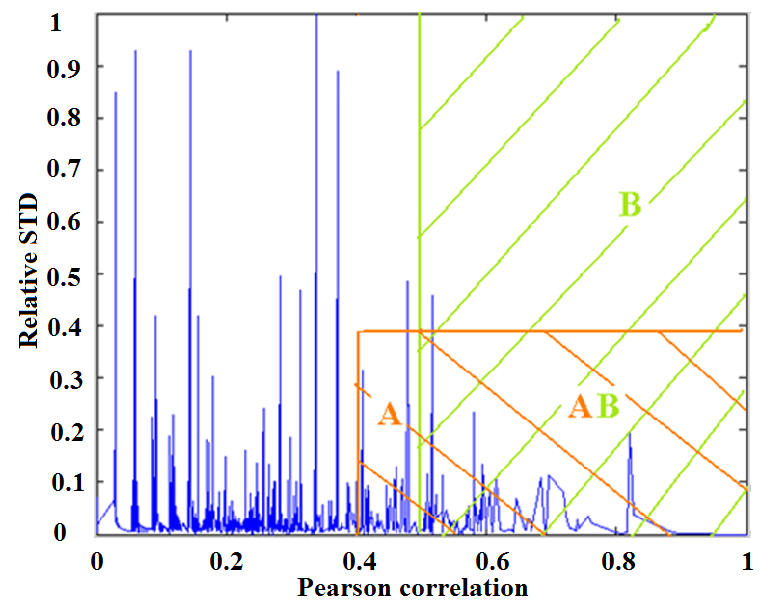


(a) (b)

**Figure 2 Diagram of Pearson’s correlation coefficient versus (a) absolute mean difference and (b) STD**; A and B are selected regions using one feature only, while AB is the region selected using both features.

Similarly, a valid candidate variable should has small standard deviation ($\mathrm{STD}$) within a group. The relationship between $\mathrm{STD}$ and Pearson’s correlation is given by Figure2 (b).

1. Rodgers JL and Nicewander WA(1988), Thirteen Ways to Look at the Correlation Coefficient. The American Statistician, 42(1): 59-66.
