# Supplementary material for: Identification of Genes for Complex Diseases Using Integrated Analysis of Multiple Types of Genomic Data
Source: PLoS One. 2012 Sep 5;7(9):e42755. doi: 10.1371/journal.pone.0042755 (PMC3434191; doi:10.1371/journal.pone.0042755)
Supplement: Supporting Material S4 — The first 50 selected expressions (with corresponding gene names) by the individual analysis of OP gene expression data. (DOCX) [file pone.0042755.s004.docx]

**Supporting Materials 4 The** **first 50 selected expressions (with corresponding gene names) by the individual analysis of OP gene expression data**

| # of expressions | Name of expressions | Name of genes | Be selected frequency |
| --- | --- | --- | --- |
| 1 | '213532_at' | *'ADAM17'* | 20 |
| 2 | '201343_at' | *'UBE2D2'* | 20 |
| 3 | '200772_x_at' | *'PTMA'* | 14 |
| 4 | '202206_at' | *'ARL4C'* | 14 |
| 5 | '202757_at' | *'COBRA1'* | 14 |
| 6 | '201219_at' | *'CTBP2'* | 13 |
| 7 | '202093_s_at' | *'PAF1'* | 13 |
| 8 | '214965_at' | *'SPATA2L'* | 12 |
| 9 | '201536_at' | *'DUSP3'* | 12 |
| 10 | '201707_at' | *'PEX19'* | 12 |
| 11 | '207688_s_at' | *'---'* | 12 |
| 12 | '201459_at' | *'RUVBL2'* | 11 |
| 13 | '202208_s_at' | *'ARL4C'* | 11 |
| 14 | '201386_s_at' | *'DHX15'* | 11 |
| 15 | '216384_x_at' | *'LOC441454 /// LOC728026 /// PTMA'* | 11 |
| 16 | '200056_s_at' | *'C1D'* | 11 |
| 17 | '208705_s_at' | *'EIF5'* | 11 |
| 18 | '217759_at' | *'TRIM44'* | 11 |
| 19 | '212136_at' | *'ATP2B4'* | 11 |
| 20 | '202660_at' | *'ITPR2'* | 11 |
| 21 | '212318_at' | *'TNPO3'* | 11 |
| 22 | '213002_at' | *'MARCKS'* | 10 |
| 23 | '201521_s_at' | *'NCBP2'* | 10 |
| 24 | '202466_at' | *'PAPD7'* | 10 |
| 25 | '206781_at' | *'DNAJC4'* | 10 |
| 26 | '203133_at' | *'SEC61B'* | 10 |
| 27 | '201114_x_at' | *'PSMA7'* | 10 |
| 28 | '222173_s_at' | *'TBC1D2'* | 10 |
| 29 | '201624_at' | *'DARS'* | 10 |
| 30 | '200600_at' | *'MSN'* | 10 |
| 31 | '218587_s_at' | *'KTELC1'* | 10 |
| 32 | '209107_x_at' | *'NCOA1'* | 10 |
| 33 | '219681_s_at' | *'RAB11FIP1'* | 10 |
| 34 | '209288_s_at' | *'CDC42EP3'* | 10 |
| 35 | '204131_s_at' | *'FOXO3'* | 10 |
| 36 | '212163_at' | *'KIDINS220'* | 10 |
| 37 | '208158_s_at' | *'OSBPL1A'* | 10 |
| 38 | '202774_s_at' | *'SFRS8'* | 10 |
| 39 | '221135_s_at' | *'ASTE1'* | 10 |
| 40 | '211004_s_at' | *'ALDH3B1'* | 10 |
| 41 | '203478_at' | *'NDUFC1'* | 10 |
| 42 | '207184_at' | *'SLC6A13'* | 10 |
| 43 | '201416_at' | *'SOX4'* | 10 |
| 44 | '218253_s_at' | *'LGTN'* | 9 |
| 45 | '215202_at' | *'LOC91316'* | 9 |
| 46 | '204057_at' | *'IRF8'* | 9 |
| 47 | '209213_at' | *'CBR1'* | 9 |
| 48 | '35148_at' | *'TJP3'* | 9 |
| 49 | '219781_s_at' | *'ZNF771'* | 9 |
| 50 | '208186_s_at' | *'LIPE'* | 9 |
