# Supplementary material for: Identification of Genes for Complex Diseases Using Integrated Analysis of Multiple Types of Genomic Data
Source: PLoS One. 2012 Sep 5;7(9):e42755. doi: 10.1371/journal.pone.0042755 (PMC3434191; doi:10.1371/journal.pone.0042755)
Supplement: Supporting Material S5 — The first 50 selected SNPs (with corresponding gene names) by the individual analysis of OP SNP data. (DOCX) [file pone.0042755.s005.docx]

**Supporting Materials 5 The first 50 selected SNPs (with corresponding gene names) by the individual analysis of OP SNP data**

| # of SNPs | Name of SNPs | Name of genes | Be selected frequency |
| --- | --- | --- | --- |
| 1 | SNP_A-1802784 | *LHX8* | 26 |
| 2 | SNP_A-1962916 | *FAM108C1* | 25 |
| 3 | SNP_A-4261886 | *EPHA4* | 23 |
| 4 | SNP_A-2094925 | *BCMO1* | 20 |
| 5 | SNP_A-1804125 | *SUMF1* | 20 |
| 6 | SNP_A-2277666 | *HHIP* | 20 |
| 7 | SNP_A-1979978 | *LOC728052* | 20 |
| 8 | SNP_A-2168731 | *CNTNAP2* | 20 |
| 9 | SNP_A-2148182 | *FAM169B* | 20 |
| 10 | SNP_A-2284158 | *ROBO1* | 20 |
| 11 | SNP_A-2206403 | *UMODL1* | 20 |
| 12 | SNP_A-1867335 | *OXA1L* | 20 |
| 13 | SNP_A-2194778 | *ZSWIM5* | 20 |
| 14 | SNP_A-2217775 | *SNAR-C1* | 20 |
| 15 | SNP_A-4269319 | *DPY19L2P4* | 20 |
| 16 | SNP_A-4283430 | *SORBS2* | 20 |
| 17 | SNP_A-2119813 | *RPL32P28* | 20 |
| 18 | SNP_A-2028580 | *SNX2* | 20 |
| 19 | SNP_A-1933731 | *GABRB3* | 20 |
| 20 | SNP_A-1961411 | *GALR1* | 20 |
| 21 | SNP_A-2207505 | *THADA* | 20 |
| 22 | SNP_A-2294955 | *LOC728292* | 20 |
| 23 | SNP_A-4279627 | *LOC100134244* | 20 |
| 24 | SNP_A-2288194 | *FAM5C* | 20 |
| 25 | SNP_A-1807556 | *LOC100131955* | 20 |
| 26 | SNP_A-2107505 | *LOC286094* | 20 |
| 27 | SNP_A-2107250 | *NECAP1* | 20 |
| 28 | SNP_A-2070934 | *RYK* | 20 |
| 29 | SNP_A-1957338 | *ELP3* | 19 |
| 30 | SNP_A-2264587 | *SLC11A1* | 19 |
| 31 | SNP_A-2222803 | *FGFR2* | 18 |
| 32 | SNP_A-2315035 | *CACNA1D* | 18 |
| 33 | SNP_A-1927545 | *ATOH7* | 18 |
| 34 | SNP_A-2124746 | *C21orf94* | 18 |
| 35 | SNP_A-2159452 | *LOC100134354* | 18 |
| 36 | SNP_A-1847227 | *ANKRD12* | 18 |
| 37 | SNP_A-2294217 | *GPC5* | 18 |
| 38 | SNP_A-1938320 | *PRKG1* | 17 |
| 39 | SNP_A-2026941 | *LRRC4C* | 17 |
| 40 | SNP_A-2311769 | *MPP7* | 17 |
| 41 | SNP_A-4304095 | *TPST1* | 17 |
| 42 | SNP_A-1903059 | *ANXA5* | 16 |
| 43 | SNP_A-1887408 | *ANO10* | 16 |
| 44 | SNP_A-2310144 | *IMMP2L* | 16 |
| 45 | SNP_A-4304165 | *C12orf50* | 15 |
| 46 | SNP_A-1790329 | *SORCS2* | 15 |
| 47 | SNP_A-1916527 | *LOC729828* | 15 |
| 48 | SNP_A-4200240 | *YWHAZ* | 15 |
| 49 | SNP_A-1933826 | *RPL7P22* | 15 |
| 50 | SNP_A-2166611 | *LOC727839* | 15 |
