# Supplementary material for: Identification of Genes for Complex Diseases Using Integrated Analysis of Multiple Types of Genomic Data
Source: PLoS One. 2012 Sep 5;7(9):e42755. doi: 10.1371/journal.pone.0042755 (PMC3434191; doi:10.1371/journal.pone.0042755)
Supplement: Supporting Material S6 — The first 50 selected SNPs and gene expressions selected (with corresponding gene names) by the joint analysis of both OP SNP and gene expression data. (DOCX) [file pone.0042755.s006.docx]

**Supporting Materials 6 The first 50 selected SNPs and gene expressions selected (with corresponding gene names) by the joint analysis of both OP SNP and gene expression data**

| # of genes | Name of genes | Name of SNPs | Name of expressions | Be selected frequency |
| --- | --- | --- | --- | --- |
| 1 | *PTMA* | SNP_A-1951766 | 200772_x_at | 20 |
| 2 | *FAM129A* | SNP_A-4243844 | 217966_s_at | 19 |
| 3 | *BBX* | SNP_A-4293605 | 213016_at | 16 |
| 4 | *DICER1* | SNP_A-1841903 | 213229_at | 15 |
| 5 | *PTMA* | SNP_A-1985640 | 200772_x_at | 14 |
| 6 | *PPIG* | SNP_A-2151832 | 208993_s_at | 14 |
| 7 | *FAM49A* | SNP_A-1910124 | 208092_s_at | 13 |
| 8 | *ATP2B4* | SNP_A-4230711 | 212136_at | 13 |
| 9 | *C1D* | SNP_A-2294317 | 200056_s_at | 12 |
| 10 | *KCNK10* | SNP_A-2292680 | 220727_at | 11 |
| 11 | *LTF* | SNP_A-2102983 | 202018_s_at | 11 |
| 12 | *CPM* | SNP_A-4299876 | 217557_s_at | 11 |
| 13 | *SCD5* | SNP_A-2079848 | 220232_at | 11 |
| 14 | *DEFA4* | SNP_A-2085306 | 207269_at | 10 |
| 15 | *KCNF1* | SNP_A-1977948 | 210263_at | 10 |
| 16 | *NUP210* | SNP_A-2288612 | 212316_at | 10 |
| 17 | *ZNF589* | SNP_A-2050055 | 219968_at | 10 |
| 18 | *PPIG* | SNP_A-2033959 | 208993_s_at | 10 |
| 19 | *PTMA* | SNP_A-2172719 | 200772_x_at | 10 |
| 20 | *PTMA* | SNP_A-4214809 | 200772_x_at | 10 |
| 21 | *PTMA* | SNP_A-1804177 | 208549_x_at | 10 |
| 22 | *PTMA* | SNP_A-1822850 | 200772_x_at | 10 |
| 23 | *ADNP2* | SNP_A-2293960 | 203321_s_at | 10 |
| 24 | *PTMA* | SNP_A-1985640 | 211921_x_at | 10 |
| 25 | *C1D* | SNP_A-2248031 | 200056_s_at | 10 |
| 26 | *ACTN1* | SNP_A-2075340 | 208636_at | 10 |
| 27 | *PTMA* | SNP_A-1934601 | 211921_x_at | 10 |
| 28 | *PTMA* | SNP_A-1934601 | 200772_x_at | 10 |
| 29 | *TRAM1* | SNP_A-4300739 | 201399_s_at | 10 |
| 30 | *PTMA* | SNP_A-2164998 | 200772_x_at | 10 |
| 31 | *PTMA* | SNP_A-1951766 | 211921_x_at | 10 |
| 32 | *DARS* | SNP_A-2269630 | 201624_at | 10 |
| 33 | *SOX4* | SNP_A-4242152 | 201417_at | 10 |
| 34 | *ATP2B4* | SNP_A-2107164 | 212136_at | 10 |
| 35 | *CDC42EP3* | SNP_A-1817778 | 209287_s_at | 10 |
| 36 | *PIAS1* | SNP_A-2014491 | 217864_s_at | 10 |
| 37 | *SRPK2* | SNP_A-2199672 | 203181_x_at | 10 |
| 38 | *TLK1* | SNP_A-4212053 | 202606_s_at | 10 |
| 39 | *SFRS8* | SNP_A-4231701 | 202774_s_at | 10 |
| 40 | *FAM49A* | SNP_A-2159804 | 208092_s_at | 10 |
| 41 | *SNCA* | SNP_A-1814017 | 207827_x_at | 10 |
| 42 | *PTMA* | SNP_A-1804177 | 200772_x_at | 10 |
| 43 | *CYP51A1* | SNP_A-1782610 | 202314_at | 10 |
| 44 | *SLC35D2* | SNP_A-4229114 | 213083_at | 10 |
| 45 | *PTMA* | SNP_A-2242102 | 200772_x_at | 10 |
| 46 | *PTMA* | SNP_A-2112017 | 200772_x_at | 10 |
| 47 | *PTMA* | SNP_A-2075882 | 200772_x_at | 10 |
| 48 | *ITPR2* | SNP_A-1881714 | 202660_at | 10 |
| 49 | *HIRA* | SNP_A-1906123 | 217427_s_at | 10 |
| 50 | *SFRS8* | SNP_A-2306550 | 202774_s_at | 10 |


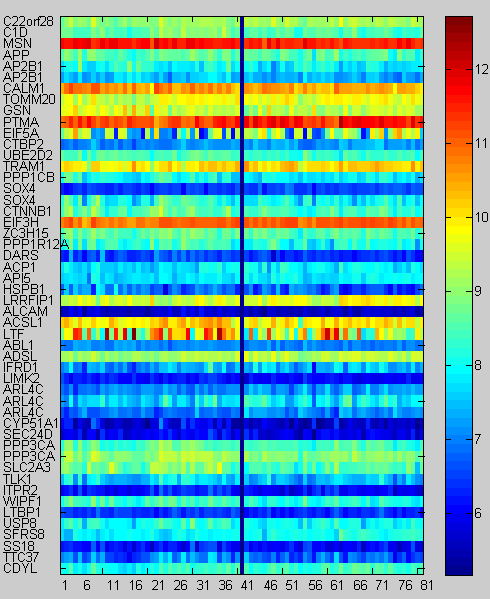


(a)


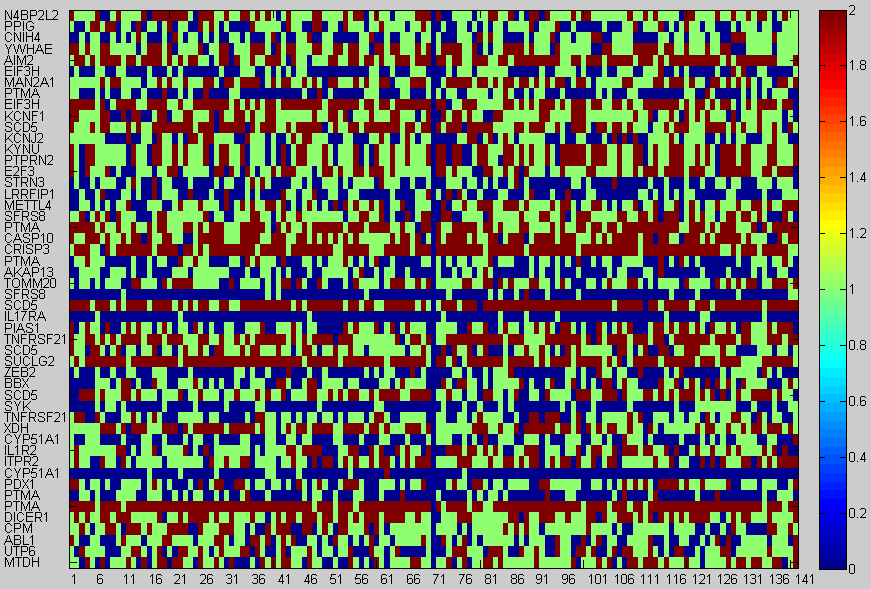


(b)

**Figure 1 The first 50 genes selected using the combined data analysis**. (a) Selected genes with the corresponding expressions from gene expression data (40/40); (b) Selected genes with the corresponding SNPs from SNP data (70/70).
